# Learning components for mixed reality mass casualty incident training: a modified Delphi study

**DOI:** 10.1186/s12909-026-08727-5

**Published:** 2026-02-05

**Authors:** Lina Gyllencreutz, Susanna Pusa, Rebecca Baxter

**Affiliations:** 1 Department of Nursing, Umeå University, Umeå, Sweden; 2 Department of Diagnostics and Intervention, Umeå University, Umeå, Sweden

**Keywords:** Delphi study, Mass casualty incident, Medical education, Mixed reality, Prehospital care, Triage

## Abstract

**Background:**

Mixed reality training encourages experiential learning to enhance skill acquisition and improve knowledge for medical first responders. The essential learning components for mixed reality innovations in the context of mass casualty incident training have yet to be explored. This modified Delphi study aimed to identify learning components for mixed reality training for mass casualty incident first responders.

**Methods:**

A modified Delphi method was undertaken comprising of three stages. In stage one, a literature search was conducted to generate statements regarding learning components for mixed reality training in mass casualty incidents. In stage two, participants rated the statements and contributed further ideas in relation to whether statements should be revised, added, or removed. In stage three, participants re-appraised their ratings in view of the group consensus. A consensus rating of 75% was set a priori. Descriptive statistical analysis was undertaken following each survey round.

**Results:**

In stage one, a total of 34 statements were derived from the literature. Eighteen experts were invited to participate. Of these, 14 provided ratings in stage two (response rate, 78%) and 8 provided ratings in stage three (response rate, 57%). Consensus was achieved for 27 of 34 statements (79%) in the first survey, and 29 of 30 statements (97%) in the second survey. Written comments from participants emphasised the importance of context in relation to the learner, the trainer, and the mixed reality system technology.

**Conclusions:**

This study identified learning components that achieved consensus for mixed reality training for mass casualty incident first responders. The learner experience, collaborative interaction, system adaptability, and training environment were important components for mixed reality training. Skills development and learner engagement were given higher priority than knowledge improvement or retention. These findings can be used to inform the planning and development of future trainings that use mixed reality modalities.

**Supplementary Information:**

The online version contains supplementary material available at 10.1186/s12909-026-08727-5.

## Background

Mixed reality (MR) combines elements of virtual reality (VR) and augmented reality (AR) [1] by incorporating digital imagery within the physical environment, where there is the possibility for both physical and digital worlds to interact and feedback [2]. MR innovations remove the existent borders that separate virtual and tactile training methodologies [3], offering a cost-effective and pragmatic alternative to medical skills training for complex situations, such as mass casualty incidents (MCI). Despite its increasing popularity, ambiguity remains regarding how to operationalize MR training in relation to learner needs and pedagogical outcomes [4].

Denolf and Kahwaji [5] define a MCI as “an event that overwhelms the local healthcare system, where the number of casualties vastly exceeds the local resources and capabilities in a short period of time” (para. 1). It is therefore essential to adequately prepare first-responders for MCIs; however, conducting full-scale MCI exercises for real-world training purposes is both time and resource intensive [6, 7]. First responders are defined by Harris et al. [8] “as a broad group that encompasses a trained cohort whose arrival to the impacted site is purposed to locate and initiate life-saving care and evacuation to the injured casualties” (p. 2). With MR technology it is possible to simulate MCIs to train medical first responders while simultaneously offering greater control over scenario and training variables [9]. As in traditional MCI simulations, MR innovations incorporate aspects including realism, collaboration, learning goals, and post-training evaluation [9]. Immersive technology-based education modalities offer possibilities for learning experiences that are interactive, engaging, and safe, while simultaneously focusing on the transfer of these skills into practice [10].

Despite its increasing popularity in the last half-decade, ambiguity remains regarding how to operationalize MR training in relation to learner needs and pedagogical approaches [11] as well as how to evaluate the quality, content and feasibility of AR curricular integration [12]. This may be due to rapid advances in MR technical capabilities, and the subsequent lag-time between the ability to implement these advances in practice, as well as differences in the comparative operational aspects of different MR systems [13]. The lack of clarity regarding learning components for MR training, and specifically MCI training, makes the comparability and interpretation of research and practice findings complex. Likewise, it is difficult to develop new MR MCI practices without first understanding the components that are viewed as essential to the approach. It is therefore necessary to establish international consensus regarding the learning components used in this line of training in order to progress the knowledge and application of MR in future innovations. This modified Delphi study aimed to identify learning components for MR training for MCI first responders. The research questions were: (a) What learning components are identified for MR training for MCI first responders? (b) Which learning components achieve expert consensus for MR training for MCI first responders?

## Methods

### Study design

A three-stage approach was undertaken for this modified Delphi study, comprising stage 1: literature search and survey statement development (November 2023 – January 2024); stage 2: consensus survey 1 (March 2024); and stage 3: revised survey statements and consensus survey 2 (April – May 2024). The Delphi method was selected for use in this study as it is suitable for systematically gathering the judgments of different experts and can be used to identify agreement and disagreement. The study protocol was not registered and no consultation with regards to the method took place. The ACcurate COnsensus Reporting Document (ACCORD) reporting guideline for consensus methods was used to report the study [14].

### Stage 1: identification of candidate statements and survey development

Study-specific survey statements were generated though a literature search and review. The literature search was conducted in December 2023 in four databases: PubMed, CINAHL SocINDEX and MEDLINE. The search string included: mixed reality training (inclusive of virtual reality, augmented reality, simulation training, and high-fidelity simulation training), and mass-casualty incidents (Supplementary File 1). By combining synonyms and related terms with the Boolean operator OR (e.g., “mixed reality” OR “virtual reality”), we ensured that studies using different terminology for similar concepts would be retrieved. Following the removal of duplicates, 271 articles underwent title and abstract screening. Of these, 32 articles progressed to full text review, and 17 were eligible for data extraction [15–32].

Next, members of the author group (LG, SP & RB) drew upon their expertise in learning and teaching principles, education implementation, and applied technology training to add and refine possible candidate statements. Thirty-eight initial statements were compiled and pilot tested among two colleagues from the authors’ university institution who had experience in emergency or disaster management and VR/MR training. Upon completion of the pilot survey the pilot testers provided feedback regarding how long it took to complete the survey, whether the survey instructions clear, whether the language easy to understand, whether the content was relevant for the study aim, if there were any challenges in completing the survey, and if they had any suggestions for improvement. Based on the pilot survey feedback, eight statements were amended and 4 statements were removed. In total, 34 statements were included in the round 1 survey (Supplementary File 2). These included statements about knowledge, interventions, assessment, outcomes, teamwork, situational awareness, decision-making, leadership, problem-solving, satisfaction, self-efficacy, stress, realism, authenticity, and debriefing. In addition, demographic questions regarding participant characteristics included respondent age, occupation, and number of years of experience relevant to MR training and/or MCI training. The survey included areas for free-text feedback where participants could provide written rationale for their response or suggestions for improvement.

### Stage 2: consensus survey round 1

Purposive convenience sampling was undertaken among experts within a European consortium who were involved in developing a MR MCI training for first responders as part of the Medical First Responder Training using a Mixed-reality Approach (MED1stMR) project [33]. The consortium included members from Greece, Austria, Germany, Spain, Sweden, and Belgium. Consortium leaders were contacted and asked to suggest three expert representatives from their organization to take part in a modified-Delphi study to identify learning priorities for MR MCI training. The criteria for experts were: experience in emergency/first responder care and/or training, and experience in development/implementation of MR MCI training. The experts were provided with information about the study and invited via email to participate. Based on the recommendation from Belton et al. [34], we aimed to achieve an expert panel of 10–20 members. Surveys were distributed electronically using ‘Survey and Report’, a web service for creating, collecting, and collating survey results.

The round 1 survey was distributed in March 2024. The experts were asked to rate 34 statements on a five-point Likert-type scale (i.e., 1 ‘not at all important’, 2 ‘somewhat not important’, 3 ‘neutral’, 4 ‘somewhat important’, 5 ‘very important’) according to the question “thinking specifically about mixed reality training that combines virtual and real-world objects/environments - how important do you think this statement is to include as a learning priority in mixed reality training for mass casualty incidents”. The experts were given two weeks to complete the survey and were sent a reminder email one week after survey distribution. Descriptive analysis of the round 1 survey responses informed the construction of statements for the round 2 survey. Agreement for consensus was defined as a rating of ‘somewhat important’ or ‘very important’. For statements that did not reach at least 75% consensus or reported an interquartile range (IQR) > 2, the author group examined the comments and proposed to either remove the statement, or amend the wording to improve comprehension. Free-text suggestions from the experts for additional statements were included in the round 2 survey.

### Stage 3: consensus survey round 2

The round 2 survey was revised based on the expert feedback from round 1 and separated into three content areas: learning priorities for MR MCI training, learner experience of MR MCI training, and learning environment for MR MCI training (Supplementary File 2). The experts who participated in the round 1 survey were asked to read a brief descriptive summary of the round 1 findings before proceeding to round 2. This included the anonymised group-level means for each of the round 1 statements, as well as their previous individual statement ratings from round 1. The round 2 survey was sent to participants in April, 2024. Participants were asked to rate the round 2 statements using the same five-point Likert-type scale. The experts were given two weeks to complete the survey and were sent a reminder email one week after survey distribution. The goal of the round 2 survey was to either (a) reach consensus on the proposed learning priorities and components, or (b) establish a requirement for a further survey. The round 2 responses were analysed descriptively to determine statements with and without consensus. Stopping criteria were specified a priori as: achieving consensus (defined as ≥ 75% agreement); statistical dispersion (assessed by IQR ≤ 2); and indication of saturation (no new items suggested by experts).

### Data analysis

Measures of central tendency were calculated for the statements during each survey round using IBM SPSS Version 28.0.1.1. Baseline demographic data were presented using percentages, means and standard deviations. Survey responses were analysed using percentages, means, standard deviations (SD), and IQRs. Free-text comments were coded by two authors independently (RB, SP) and analysed thematically. Initial codes were developed inductively, then grouped into categories that reflected the wording of the statements (cf. [35]).

## Results

A total of 18 experts were invited to participate in the study, of whom 14 responded to the round 1 survey (response rate, 78%; Table 1). The experts who responded to the round 1 survey were located in Belgium (*n* = 2), Sweden (*n* = 3), Greece (*n* = 2), Austria (*n* = 2), Germany (*n* = 2), and Spain (*n* = 3). Of the 14 participants who completed the round 1 survey, 8 responded to the round 2 survey (response rate, 57%). The experts who responded to the round 2 survey were located in Belgium (*n* = 1), Sweden (*n* = 3), Greece (*n* = 1), Austria (*n* = 2), and Spain (*n* = 1). In both survey rounds the majority of participants reported having over 11 years of experience in MR and/or MCI emergency education and/or clinical practice.

**Table 1:** Participants’ demographic information

Participants	Survey Round 1 (*N* = 14)	Survey Round 2 (*N* = 8)
Age	Mean, 43.86 (min 34, max 52)	Mean 44.88 (min 34, max 52)
Gender		
Female	35.7% (*n* = 5)	50% (*n* = 4)
Male	64.3% (*n* = 9)	50% (*n* = 4)
Number of years of experience		
6–10 years	21.4% (*n* = 3)	25% (*n* = 2)
11 + years	78.6% (*n* = 11)	75% (*n* = 6)
Self-reported professional title^a^		
Registered Nurse/Nurse Specialist	6	4
Physician/Specialist Physician	5	2
Paramedic	1	1
Medical Researcher/Clinical Researcher	4	1
Engineer/Clinical Instructor	1	-

^a^some participants held multiple roles in addition to their main professional job title

In round 1, consensus was reached for 27 of 34 statements (79.4%; Table 2). The mean scores ranged 3.5–4.9 (SD, 0.36–1.50) and the IQR ranged 0–3 (Supplementary File 2). The 7 statements that did not reach consensus in round 1 were removed from the round 2 survey. Three statements were added to the round 2 survey based on participants’ written feedback to the round 1 survey. The round 2 mean scores ranged 3.6–5.0 (SD, 0–0.90) and the IQR ranged 0–2 (Supplementary File 2). In round 2, consensus was reached for 29 of 30 statements (96.7%; Table 2).

**Table 2:** MR MCI training component statements with or without consensus

Statements	Consensus achieved^a^	
	Survey Round 1	Survey Round 2
Learning priorities for MR MCI training		
Apply theoretical knowledge during trainings	Yes	No
Improve knowledge during trainings (assessed via metrics)	No	-
Retain knowledge between trainings	Yes	Yes
Identify main clinical problem (for the patient)	No	-
Identify the appropriate clinical intervention (for the patient)	Yes	Yes
Develop life-saving clinical skills (e.g., open airway, haemorrhage control)	No	-
Assess primary triage category	Yes	Yes
Complete timely primary triage (time to correct triage)	Yes	Yes
Complete timely clinical intervention (time to correct intervention)	No	-
Achieve a positive patient outcome (e.g., survival)	Yes	Yes
Develop communication between learner and patients	No	-
Develop communication between learner and team members	Yes	Yes
Develop teamwork abilities (collaboration among learners)	Yes	Yes
Develop situational awareness	Yes	Yes
Develop decision-making skills	Yes	Yes
Develop disaster coordination response skills (relative to role)	Yes	Yes
Analyse complex situations	Yes	Yes
Develop leadership skills	Yes	Yes
Develop problem-solving skills	Yes	Yes
Develop preparedness for responding to MCI	Yes	Yes
Develop performance metrics in repeated trainings (e.g., speed, accuracy)	Yes	Yes
Coordinate with other first responder organizations (e.g., police, fire brigade)^b, c^	-	Yes
Learner experience of MR MCI training		
Achieve learner satisfaction with the training	Yes	Yes
Achieve learner immersion in the training	Yes	Yes
Develop learner confidence	Yes	Yes
Develop learner self-efficacy	Yes	Yes
Obtain feedback on learner performance after the training	Yes	Yes
Learning environment for MR MCI training		
Simulate stress	Yes	Yes
Simulate danger	Yes	Yes
Use tangible physical objects (e.g., torniquet)	No	-
Move around the physical space	Yes	Yes
Move between virtual and physical environments	No	-
Align the scenario and predetermined learning objectives	Yes	Yes
Achieve scenario realism (how the training looks compared to a real MCI)	Yes	Yes
Achieve scenario authenticity (similarity of the training experience to a real MCI)	Yes	Yes
Train different scenarios (include more than one scenario in the training)^b^	-	Yes
Debrief after each scenario^b^	-	Yes

^a^Consensus ≥75%

^b^statement added in survey round 2 based on participant feedback

^c^missing response *n* =1

Certain clinical skills and knowledge aspects were rated by experts as less important for MR MCI training. Regarding learner experience, all experts agreed that learner satisfaction, immersion, confidence, self-efficacy and feedback were very important or somewhat important components for MR MCI training. The learning environment statements achieved unanimous consensus in both survey rounds, indicating that experts viewed these as integral components of MR MCI training. Statements regarding the use of tangible physical objects and movement between the MR and VR environments did not reach consensus in round 1, possibly indicating that MR training outcomes require a more seamless integration of these aspects instead of an explicit separation. However, considerations regarding the necessity of MR over VR features were not explicitly defined. Despite achieving consensus in round 1, during round 2 experts indicated that ‘achieving a positive outcome (patient survival)’ was too difficult to judge as a learning outcome in MR as triage and first treatment could still lead to a so-called negative patient outcome. Other learning outcomes that were included after feedback from round 1 were affirmed in round 2, including the additional component of coordinating with other emergency organizations.

The free-text comments and suggestions from participants in round 1 and 2 emphasised the importance of context in relation to the learner, the trainer, and the technical possibilities of the MR system. Participants noted that learner performance would likely improve between trainings, so the learning goals/objectives should be scored with consideration given to the number of times they had trained using specific scenarios or systems. It was also noted that learning outcomes and components had to be balanced in relation to learner experience depending on the chosen MR MCI scenario and/or system design.

## Discussion

This study aimed to identify learning components for MR MCI training for first responders using a modified-Delphi method. Expert consensus was achieved for 29 statements. The consensus statements highlight the importance of elucidating the learning priorities, the learner experience, and the learning environment in MR MCI training. The results revealed differences compared with training objectives used by other systems or traditions, indicating that MR is a unique modality that requires further explication. Statements that did not achieve consensus in the first or second rounds mainly pertained to knowledge improvement and retention, patient communication, and the physical-virtual environment. These findings emphasize the need for a common understanding regarding MR MCI training components through improved awareness and collaborative discussions involving key stakeholders.

Situated learning theory emphasises that learning is a socially situated practice that does not prioritize internalized individualistic cerebral practices, instead the focus is on the interactions and exchanges that occur socially via dynamic participation [36]. In this way, knowledge is viewed as a natural product of social activity anchored within a particular context of meaningful systems and relations [36]. The statements that achieved consensus for MR MCI training were centred around the learner experience, but also recognised the importance of collaboration, teamwork, and interaction. This included learning components that emphasized exchanges between the learner and the ‘patient’, the learner and other first responder organisations, the learner and other learners, and between the learner and the trainer. This appears to support previous calls to focus on ensuring alignment between learning objectives and functional aspects within simulations, instead of concentrating on the physical or structural properties of simulations [37]. While situated learning theory was not used as a guiding framework in the design of this study, it can serve as an interpretive lens to help understand how learning occurs in context-rich environments. These findings strongly indicate that both the physical and social environments should be considered in the design of MR MCI training systems and components to best support situated learning in this unique context.

Simulation scenarios encourage clinical skill development through exposure, practice and reflection [38]. When constructing simulation-based education for health professionals it is important to maintain a level of fidelity to the experiential components and the environment in order to promote learner engagement [37]. However, physical resemblance and functional task alignment can be rated as low or high by learners depending on the ways in which they experience visual, tactile or functional aspects of a simulation, as well as factors relating to the individual, the learning objectives, and the learning setting [37]. From a situated learning theory viewpoint, learning comprises interaction, participation, cooperation and collaboration to shape the learner’s identity. The constantly changing systems of relations shape the experience and the learner thus emerges with new and different perspectives [36]. Integration of immersive learning technologies and simulation-based modalities therefore must navigate the reality-virtuality continuum [38] in a way that encompasses the socio-cultural context of healthcare training. The consensus statements reflected that while fidelity aspects such as realism and authenticity were important, the use of tangible objects and movement between the physical and virtual ‘worlds’ was not found to be relevant in relation to the overall MR MCI learning components. Thus, essential learning components for MR MCI training innovations should not aim to echo reality, but should instead prioritise replication of the conditions needed to learn.

The findings from this study revealed that MR MCI training must be adapted to achieve specific components related to learning priorities, learner experience and the learning environment. Coherence in scenarios relates to the extent to which the technology is able to reflect the real-world experience [39]. The use of headsets in VR/AR facilitates visual immersion and high fidelity in scenarios that would otherwise be near-impossible to recreate in a simulated environment [38]. A recent systematic review found that high-fidelity VR MCI simulations provided a safe and realistic environment to repeatedly prepare first responders for MCI, but that physiological demands and levels of stress were not as high compared to live simulation [40]. Trainee perceptions of virtual simulation for MCI training remain mixed, with virtual and e-learning technologies viewed by some trainees as supplements to other MCI training formats and methodologies [41]. This emphasises the importance of evaluating the fidelity of both structure and function in simulation-based training [37]. As the experts noted in their free-text comments, it is necessary to consider and incorporate context in relation to the learner, the trainer, the technical possibilities of the system. In this way, system-level flexibility and adaptability were identified as important components in MR MCI training that require consistent (re)evaluation in relation to the learner experience and learning outcomes.

### Limitations

Although the experts represented different stakeholder groups across Europe, the sample size was relatively small and dropout was observed between survey round 1 and 2; however, the experts who participated in this study represented a range of clinical and educational specialities and the number of participants fell within the recommended range for this method [34]. It is not possible to draw broader conclusions regarding the opinions of other stakeholders, such as MR technicians or software developers. This could be an avenue for further research. We acknowledge that the experts involved in this study were familiar with the MED1stMR training system and may have had this system in mind when answering the survey which could have influenced their responses. This could be viewed as an advantage as it guaranteed that the participants had expertise in the relevant subject matter. It should also be noted that achieving consensus can be more challenging when a common first language is not shared. The survey language was English, which was not the first language for some of the experts. Translating the survey into other languages was beyond the scope of this study.

The decision to use an ‘importance scale’ rather than a ‘necessity scale’ allowed experts to express nuanced opinions about each potential learning component according to its perceived application for MR MCI first responder training without forcing a judgement about necessity [42]. This may have resulted in the inclusion of learning components deemed important but not necessary, offering an interesting direction for future research. Consensus was defined a priori as ≥ 75% agreement, consistent with recommendations in Delphi methodology literature [34]. While setting higher thresholds could indicate stronger consensus, they can be overly restrictive and impractical for studies aiming to identify broadly relevant components. Likewise, stricter IQR thresholds could indicate greater stability [34]. However, setting an IQR of ≤ 2 allowed us to retain components with broad relevance to the study aim without excluding them prematurely. Lastly, due to the dearth of MR MCI literature the initial survey statements were developed from literature that included traditional simulation training as well as AR, VR and XR concepts in a variety of medical educational settings. This provided an important theoretical foundation to the statements, and the subsequent expert (non)consensus confirmed their relevance for the content area of MR MCI training. These concepts should be continually examined as new research with a specific focus on MR training emerges.

## Conclusions

This study established consensus on learning components for first responder MR MCI training. Expert consensus demonstrated that the learner experience, collaborative interaction, system adaptability, and training environment were important components. Skills development and learner engagement were given higher priority than knowledge improvement or retention. These consensus statements can be used by clinicians, educators, and policymakers who are involved in the planning, delivery and evaluation of MR MCI trainings. Further research is needed to translate these components into practice and evaluate the effectiveness of their application.

## Supplementary Information

Supplementary Material 1.

Supplementary Material 2.

## Data Availability

The data are not publicly available as the study participants did not give consent for their individual data to be shared in the public forum.

## Abbreviations

ACCORD: ACcurate COnsensus Reporting Document
IQR: Interquartile Range
MR: Mixed Reality
VR: Virtual Reality
AR: Augmented Reality
MCI: Mass Casualty Incidents
MED1stMR: Medical First Responder Training using a Mixed-reality Approach
