# Supplementary material for: Learning components for mixed reality mass casualty incident training: a modified Delphi study
Source: BMC Med Educ. 2026 Feb 5;26:338. doi: 10.1186/s12909-026-08727-5 (PMC12930572; doi:10.1186/s12909-026-08727-5)
Supplement: Supplementary file 1 — Supplementary Material 1. [file 12909_2026_8727_MOESM1_ESM.docx]

**Search strategy**

| Database | Search |
| --- | --- |
| CINAHL | AB ( "mixed-realit*" OR "mixed realit*" OR "virtual-realit*" OR "virtual realit*" OR "augmented-realit*" OR "augmented realit*" OR "High-fidelity simulation training" OR "simulation training" ) AND AB ( "mass-casualty" OR "mass casualty" OR "mass-casualty incident*" OR "mass casualty incident*" OR "major incident*" OR "major accident*" OR "catastrophe*" OR disaster* OR "disaster medicine" OR "emergency medical service*" OR "medical emergency response*" OR "emergency service*" OR "first responder*" OR "first-responder*" ) AND AB ( intervention* OR training* OR program* OR curricul* OR exercise* OR practice* OR simulation* OR experience* OR scenario* OR learn* OR education* OR workshop* OR skill* OR goal* OR tool* OR component* OR competenc* )  TI ( "mixed-realit*" OR "mixed realit*" OR "virtual-realit*" OR "virtual realit*" OR "augmented-realit*" OR "augmented realit*" OR "High-fidelity simulation training" OR "simulation training" ) AND TI ( "mass-casualty" OR "mass casualty" OR "mass-casualty incident*" OR "mass casualty incident*" OR "major incident*" OR "major accident*" OR "catastrophe*" OR disaster* OR "disaster medicine" OR "emergency medical service*" OR "medical emergency response*" OR "emergency service*" OR "first responder*" OR "first-responder*" ) AND TI ( intervention* OR training* OR program* OR curricul* OR exercise* OR practice* OR simulation* OR experience* OR scenario* OR learn* OR education* OR workshop* OR skill* OR goal* OR tool* OR component* OR competenc* ) |
| PubMed | (("mixed-realit*"[Title/Abstract] OR "mixed realit*"[Title/Abstract] OR "virtual-realit*"[Title/Abstract] OR "virtual realit*"[Title/Abstract] OR "augmented-realit*"[Title/Abstract] OR "augmented realit*"[Title/Abstract] OR "High-fidelity simulation training"[Title/Abstract] OR "simulation training"[Title/Abstract]) AND ("mass-casualty"[Title/Abstract] OR "mass casualty"[Title/Abstract] OR "mass-casualty incident*"[Title/Abstract] OR "mass casualty incident*"[Title/Abstract] OR "major incident*"[Title/Abstract] OR "major accident*"[Title/Abstract] OR "catastrophe*"[Title/Abstract] OR disaster*[Title/Abstract] OR "disaster medicine"[Title/Abstract] OR "emergency medical service*"[Title/Abstract] OR "medical emergency response*"[Title/Abstract] OR "emergency service*"[Title/Abstract] OR "first responder*"[Title/Abstract] OR "first-responder*"[Title/Abstract])) AND (intervention*[Title/Abstract] OR training*[Title/Abstract] OR program*[Title/Abstract] OR curricul*[Title/Abstract] OR exercise*[Title/Abstract] OR practice*[Title/Abstract] OR simulation*[Title/Abstract] OR experience*[Title/Abstract] OR scenario*[Title/Abstract] OR learn*[Title/Abstract] OR education*[Title/Abstract] OR workshop*[Title/Abstract] OR skill*[Title/Abstract] OR goal*[Title/Abstract] OR tool*[Title/Abstract] OR component*[Title/Abstract] OR competenc*[Title/Abstract]) Filters: English |
| SocIndex | TX ( "mixed-realit*" OR "mixed realit*" OR "virtual-realit*" OR "virtual realit*" OR "augmented-realit*" OR "augmented realit*" OR "High-fidelity simulation training" OR "simulation training" ) AND TX ( "mass-casualt*" OR "mass casualt*" OR "mass-casualty incident*" OR "mass casualty incident*" OR "major incident*" OR "major accident*" OR "catastrophe*" OR disaster* OR "disaster medicine" OR "emergency medical service*" OR "medical emergency response*" OR "emergency service*" OR "first responder*" OR "first-responder*" ) AND TX ( intervention* OR training* OR program* OR curricul* OR exercise* OR practice* OR simulation* OR experience* OR scenario* OR learn* OR education* OR workshop* OR skill* OR goal* OR tool* OR component* OR competenc* ) |
| MEDLINE | AB ( "mixed-realit*" OR "mixed realit*" OR "virtual-realit*" OR "virtual realit*" OR "augmented-realit*" OR "augmented realit*" OR "High-fidelity simulation training" OR "simulation training" ) AND AB ( "mass-casualt*" OR "mass casualt*" OR "mass-casualty incident*" OR "mass casualty incident*" OR "major incident*" OR "major accident*" OR "catastrophe*" OR disaster* OR "disaster medicine" OR "emergency medical service*" OR "medical emergency response*" OR "emergency service*" OR "first responder*" OR "first-responder*" ) AND AB ( intervention* OR training* OR program* OR curricul* OR exercise* OR practice* OR simulation* OR experience* OR scenario* OR learn* OR education* OR workshop* OR skill* OR goal* OR tool* OR component* OR competenc* ) |
