# Supplementary material for: Learning components for mixed reality mass casualty incident training: a modified Delphi study
Source: BMC Med Educ. 2026 Feb 5;26:338. doi: 10.1186/s12909-026-08727-5 (PMC12930572; doi:10.1186/s12909-026-08727-5)
Supplement: Supplementary file 2 — Supplementary Material 2. [file 12909_2026_8727_MOESM2_ESM.docx]

**Delphi survey statements and consensus**

| **Survey statement** | **Survey round 1** | | | | **Survey round 2** | | | |
| --- | --- | --- | --- | --- | --- | --- | --- | --- |
|  | **Mean** | **SD** | **%** | **IQR** | **Mean** | **SD** | **%** | **IQR** |
| **Learning priorities for MR MCI training** | | | | | | | | |
| Apply theoretical knowledge during trainings | 4.07 | 0.92 | 78.6 | 1 | 3.71 | 0.76 | 62.5 | 1 |
| Improve knowledge during trainings (assessed via metrics) | 3.93 | 1.41 | 64.3 | 2 | - | - | - | - |
| Retain knowledge between trainings | 4.21 | 0.80 | 92.8 | 1 | 4.14 | 0.69 | 87.5 | 1 |
| Identify main clinical problem (for the patient) | 4.00 | 1.11 | 71.5 | 2 | - | - | - | - |
| Identify the appropriate clinical intervention (for the patient) | 4.50 | 0.94 | 85.7 | 1 | 4.57 | 0.54 | 100 | 1 |
| Develop life-saving clinical skills (e.g., open airway, haemorrhage control) | 3.50 | 1.50 | 57.1 | 3 | - | - | - | - |
| Assess primary triage category | 4.71 | 0.47 | 100 | 1 | 5.0 | 0.0 | 100 | 0 |
| Complete timely primary triage (time to correct triage) | 4.43 | 0.65 | 92.9 | 1 | 4.86 | 0.38 | 100 | 0 |
| Complete timely clinical intervention (time to correct intervention) | 4.00 | 1.04 | 64.3 | 2 | - | - | - | - |
| Achieve a positive patient outcome (e.g., survival) | 4.14 | 1.17 | 78.6 | 1 | 4.14 | 0.90 | 75 | 2 |
| Develop communication between learner and patients | 3.93 | 1.07 | 71.4 | 2 | - | - | - | - |
| Develop communication between learner and team members | 4.79 | 0.43 | 100 | 0 | 5.0 | 0.0 | 100 | 0 |
| Develop teamwork abilities (collaboration among learners) | 4.71 | 0.47 | 100 | 1 | 5.0 | 0.0 | 100 | 0 |
| Develop situational awareness | 4.57 | 0.65 | 92.9 | 1 | 4.86 | 0.38 | 100 | 0 |
| Develop decision-making skills | 4.86 | 0.36 | 100 | 0 | 5.0 | 0.0 | 100 | 0 |
| Develop disaster coordination response skills (relative to role) | 4.64 | 0.75 | 85.7 | 0 | 4.86 | 0.38 | 100 | 0 |
| Analyse complex situations | 4.21 | 0.98 | 78.6 | 1 | 4.57 | 0.54 | 100 | 1 |
| Develop leadership skills | 4.50 | 0.86 | 92.9 | 1 | 4.71 | 0.49 | 100 | 1 |
| Develop problem-solving skills | 4.50 | 0.52 | 100 | 1 | 4.71 | 0.49 | 100 | 1 |
| Develop preparedness for responding to MCI | 4.79 | 0.43 | 100 | 0 | 5.0 | 0.0 | 100 | 0 |
| Develop performance metrics in repeated training scenarios (e.g., speed, accuracy) | 3.93 | 1.00 | 78.6 | 1 | 3.57 | 0.79 | 75 | 1 |
| Coordinate with other first responder organizations (e.g., police, fire brigade)^b, c^ | - | - | - | - | 4.71 | 0.76 | 85.7 | 0 |
| **Learner experience of MR MCI training** | | | | | | | | |
| Achieve learner satisfaction with the training | 4.29 | 0.61 | 92.9 | 1 | 4.29 | 0.49 | 100 | 1 |
| Achieve learner immersion in the training | 4.64 | 0.50 | 100 | 1 | 4.43 | 0.54 | 100 | 1 |
| Develop learner confidence | 4.64 | 0.50 | 100 | 1 | 4.86 | 0.38 | 100 | 0 |
| Develop learner self-efficacy | 4.57 | 0.65 | 92.9 | 1 | 4.43 | 0.54 | 100 | 1 |
| Obtain feedback on learner performance after the training | 4.71 | 0.47 | 100 | 1 | 4.86 | 0.38 | 100 | 0 |
| **Learning environment for MR MCI training** | | | | | | | | |
| Simulate stress | 4.57 | 0.65 | 92.9 | 1 | 4.57 | 0.54 | 87.5 | 1 |
| Simulate danger | 4.36 | 0.75 | 85.7 | 1 | 4.43 | 0.54 | 87.5 | 1 |
| Use tangible physical objects (e.g., torniquet) | 3.93 | 1.27 | 71.5 | 2 | - | - | - | - |
| Move around the physical space | 4.57 | 0.51 | 100 | 1 | 4.71 | 0.49 | 87.5 | 1 |
| Move between virtual and physical environments | 3.93 | 1.27 | 71.5 | 2 | - | - | - | - |
| Align the scenario and predetermined learning objectives | 4.43 | 0.65 | 92.9 | 1 | 4.57 | 0.54 | 100 | 1 |
| Achieve scenario realism (how the training looks compared to a real MCI) | 4.57 | 0.51 | 100 | 1 | 4.71 | 0.49 | 87.5 | 1 |
| Achieve scenario authenticity (how similar the training experience is to a real MCI) | 4.57 | 0.85 | 92.9 | 1 | 4.57 | 0.54 | 100 | 1 |
| Train different scenarios (include more than one scenario in the training)^b^ | - | - | - | - | 4.86 | 0.38 | 87.5 | 0 |
| Debrief after each scenario^b^ | - | - | - | - | 5.0 | 0.0 | 100 | 0 |

^a Consensus was reached if agreed by ≥75% of participants; b statement added in round 3 based on participant feedback; c percentage adjusted due to missing response in R2 (n=1).^
